# Fecal Viral Diversity of Captive and Wild Tasmanian Devils Characterized Using Virion-Enriched Metagenomics and Metatranscriptomics

**DOI:** 10.1128/JVI.00205-19

**Published:** 2019-05-15

**Authors:** Rowena Chong, Mang Shi, Catherine E. Grueber, Edward C. Holmes, Carolyn J. Hogg, Katherine Belov, Vanessa R. Barrs

**Affiliations:** a School of Life and Environmental Sciences, University of Sydney, Sydney, New South Wales, Australia; b Marie Bashir Institute for Infectious Diseases and Biosecurity, Sydney Medical School, University of Sydney, Sydney, New South Wales, Australia; c School of Life and Environmental Sciences and Sydney Medical School, Charles Perkins Centre, University of Sydney, Sydney, New South Wales, Australia; d San Diego Zoo Global, San Diego, California, USA; e Sydney School of Veterinary Science, University of Sydney, Sydney, New South Wales, Australia; Instituto de Biotecnologia/UNAM

**Keywords:** carnivore, endangered species, evolutionary biology, marsupial, microbial ecology, microbiome, virus

## Abstract

The Tasmanian devil is an endangered carnivorous marsupial threatened by devil facial tumor disease (DFTD). While research on DFTD has been extensive, little is known about viruses in devils and whether any are of potential conservation relevance for this endangered species. Using both metagenomics based on virion enrichment and sequence-independent amplification (virion-enriched metagenomics) and metatranscriptomics based on bulk RNA sequencing, we characterized and compared the fecal viromes of captive and wild devils. A total of 54 fecal samples collected from two captive and four wild populations were processed for virome characterization using both approaches. In total, 24 novel marsupial-related viruses, comprising a sapelovirus, astroviruses, rotaviruses, picobirnaviruses, parvoviruses, papillomaviruses, polyomaviruses, and a gammaherpesvirus, were identified, as well as known mammalian pathogens such as rabbit hemorrhagic disease virus 2. Captive devils showed significantly lower viral diversity than wild devils. Comparison of the two virus discovery approaches revealed substantial differences in the number and types of viruses detected, with metatranscriptomics better suited for RNA viruses and virion-enriched metagenomics largely identifying more DNA viruses. Thus, the viral communities revealed by virion-enriched metagenomics and metatranscriptomics were not interchangeable and neither approach was able to detect all viruses present. An integrated approach using both virion-enriched metagenomics and metatranscriptomics constitutes a powerful tool for obtaining a complete overview of both the taxonomic and functional profiles of viral communities within a sample.

**IMPORTANCE** The Tasmanian devil is an iconic Australian marsupial that has suffered an 80% population decline due to a contagious cancer, devil facial tumor disease, along with other threats. Until now, viral discovery in this species has been confined to one gammaherpesvirus (dasyurid herpesvirus 2 [DaHV-2]), for which captivity was identified as a significant risk factor. Our discovery of 24 novel marsupial-associated RNA and DNA viruses, and that viral diversity is lower in captive than in wild devils, has greatly expanded our knowledge of gut-associated viruses in devils and provides important baseline information that will contribute to the conservation and captive management of this endangered species. Our results also revealed that a combination of virion-enriched metagenomics and metatranscriptomics may be a more comprehensive approach for virome characterization than either method alone. Our results thus provide a springboard for continuous improvements in the way we study complex viral communities.

## Abstract (precis)

The Tasmanian devil is an iconic Australian marsupial that has suffered an 80% population decline due to a contagious cancer, devil facial tumor disease, along with other threats. Until now, viral discovery in this species has been confined to one gammaherpesvirus (dasyurid herpesvirus 2 [DaHV-2]), for which captivity was identified as a significant risk factor. Our discovery of 24 novel marsupial-associated RNA and DNA viruses, and that viral diversity is lower in captive than in wild devils, has greatly expanded our knowledge of gut-associated viruses in devils and provides important baseline information that will contribute to the conservation and captive management of this endangered species. Our results also revealed that a combination of virion-enriched metagenomics and metatranscriptomics may be a more comprehensive approach for virome characterization than either method alone. Our results thus provide a springboard for continuous improvements in the way we study complex viral communities.

## INTRODUCTION

The Tasmanian devil (Sarcophilus harrisii) is the world’s largest extant carnivorous marsupial and is found in the wild only on the island state of Tasmania, Australia. As this animal is predominately a scavenger, its diet largely comprises carrion of mammals, including wallabies, possums, and kangaroos, although it may also consume and digest fish, insects, fruit, and vegetation (1, 2). Listed as endangered, the Tasmanian devil is facing the threat of extinction due to a contagious cancer, devil facial tumor disease (DFTD1 and -2), that has caused drastic (77%) declines in wild devil populations since its discovery in 1996 (3). In an attempt to save the species from extinction, an insurance population was established in 2006 to supplement wild populations at risk of population crashes (4, 5). While extensive research has focused on DFTD, susceptibility to DFTD, and devil genetic diversity (6–10), understanding of other disease threats to devils remains limited. The gut microbiome has been characterized (11), but virological studies are limited to the identification of a single gammaherpesvirus (dasyurid herpesvirus 2 [DaHV-2]), for which captivity was identified as a significant risk factor (12). Characterization of viral diversity among Tasmanian devils is an essential step to improve understanding of host-microbe relationships, and for conservation management of the species. Comparative analysis of marsupial viruses with those from diverse vertebrate hosts, including eutherian mammals, birds, and other vertebrates, will also provide a deeper understanding of the phylogenetic history of the viruses infecting this evolutionary unique group of mammals (13, 14).

The most widely used method for studying viral metagenomics relies on the enrichment of virions and sequence-independent amplification prior to sequencing (15–17). Removal of nonviral genomic host and bacterial nucleic acids is often necessary for the detection of low-titer viruses (17, 18). More recently, the use of RNA sequencing of total non-rRNA from environmental samples gave rise to viral metatranscriptomics, which has been successfully applied to characterize the viromes of diverse invertebrate and vertebrate species (13, 19, 20). To our knowledge, no studies to date have directly compared these two approaches to virome characterization, although doing so would allow us to understand the detection capabilities and biases associated with different nucleic acid extraction and sample treatment methods.

We characterized the fecal virome of wild and captive Tasmanian devil using both metagenomics based on virion enrichment and sequence-independent amplification (here “virion-enriched metagenomics”) and metatranscriptomics based on RNA sequencing of total non-rRNA (here “metatranscriptomics”). Our objectives were (i) to comprehensively characterize the fecal virome of Tasmanian devils, comparing those of wild and captive devils, and (ii) to compare the two virome characterization approaches, highlighting their advantages and potential challenges.

(This article was submitted to an online preprint archive [21].)

## RESULTS

### Overview of the devil virome.

**FIG 1:**
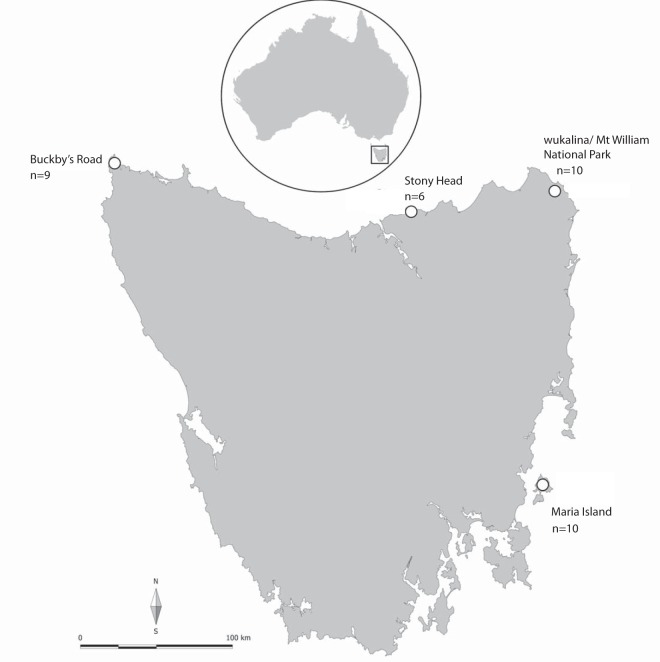
Map of Tasmania, Australia, showing the four wild sampling sites. The number of fecal samples in each location pool is also indicated. The two captive sampling sites are located on mainland Australia and are designated zoo A and zoo B, with 9 and 10 unique fecal samples in each pool, respectively. (Courtesy of A. V. Lee; reproduced with permission.)

**FIG 2:**
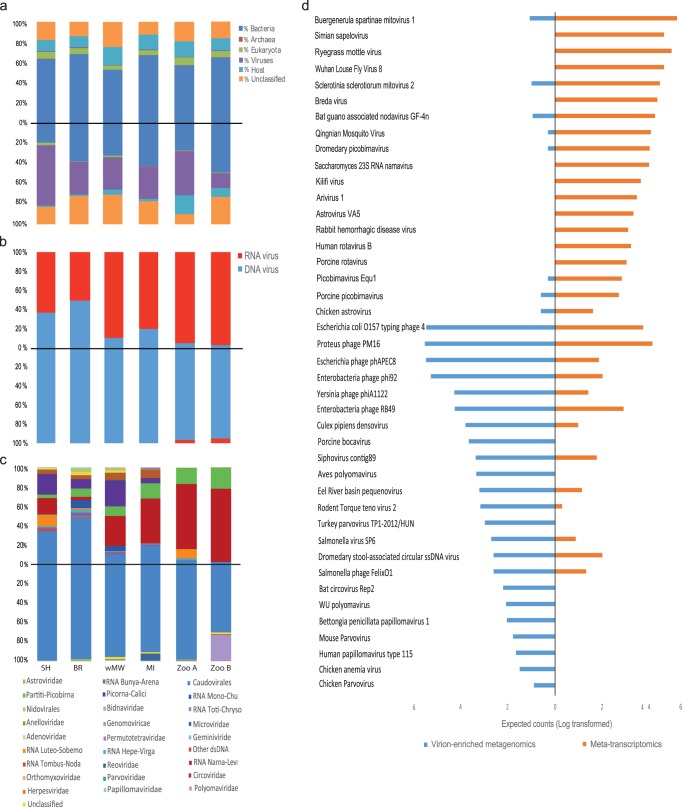
Overview of the Tasmanian devil fecal virome characterized by metatranscriptomics and virion-enriched metagenomics. Sequencing library/sampling site are represented at the bottom of the bar charts by SH for Stony Head, BR for Buckbys Road, wMW for wukalina/Mt William National Park, MI for Maria Island, and Zoo A and Zoo B for the two captive populations. (a) Proportions of all sequence reads in metatranscriptomics (top) and virion-enriched metagenomics (bottom) categorized as belonging to bacteria, eukaryotes, archaea, viruses, and hosts or unidentified. (b) Proportions of RNA and DNA viruses detected by metatranscriptomics (top) and virion-enriched metagenomics (bottom). (c) Virome composition and the proportions of viral groups in metatranscriptomics (top) and virion-enriched metagenomics (bottom). (d) Estimated counts as calculated by RSEM (all six sequencing libraries from both virion-enriched metagenomics and metatranscriptomics combined and log transformed) to a selection of viruses, showing the differences in viruses detected by virion-enriched metagenomics and metatranscriptomics.

We characterized the fecal virome of six pools consisting of a total of 54 unique fecal samples collected from 54 individual Tasmanian devils from four wild sites and two captive sites (see Materials and Methods and Fig. 1) using both metatranscriptomics and virion-enriched metagenomics approaches. Metatranscriptomic sequencing resulted in 128 to 140 million reads per pool (793,038,436 reads in total), which were assembled *de novo* into 196,919 to 358,327 contigs (Table 1). BLAST analyses of sequence reads from the metatranscriptomic protocol revealed large proportions of reads from *Bacteria* (55.11 to 67.77%) and only 4.32 to 7.88% from *Eukarya*. Mapping reads to the Tasmanian devil genome revealed that 10.75 to 18.99% of reads originated from the host. The percentage of reads related to *Archaea* was less than 0.02%, and the value for viruses was between 0.68 and 1.16% (Fig. 2a).

**TABLE 1:** Library information of metatranscriptomics and virion-enriched metagenomics in the present study

Location	C^*a*^/W^*b*^	Metatranscriptomics		Virion-enriched metagenomics	
		No. of reads	No. of contigs	No. of reads	No. of contigs
Zoo A	C	139,827,866	219,496	26,704,842	281,857
Zoo B	C	128,149,408	196,919	33,002,790	267,536
Maria Island	W	129,783,390	261,483	42,856,850	153,804
Buckbys Road	W	128,589,556	358,327	42,602,752	84,646
wukalina/Mt William National Park	W	129,981,856	264,205	48,911,728	313,154
Stony Head	W	136,706,360	325,769	43,095,274	49,812
Total	793,038,436	1,626,199	237,174,236	1,150,809	

a C, captive.

b W, Wild.

Virion-enriched metagenomics resulted in 26 to 49 million reads per pool (237,174,236 reads in total), which were assembled *de novo* into 49,813 to 313,354 contigs (Table 1). Compared to metatranscriptomics, the virion-enriched metagenomics protocol resulted in smaller proportions of nonviral nucleic acids (host and bacterial) and hence enriched viral nucleic acids (Fig. 2a). The proportion of viral reads in virion-enriched metagenomics varied from 14.69 to 60.02%, while the proportions of reads mapped to nonviral components were 17 to 49.46% for *Bacteria*, 0.17 to 2.42% for *Eukarya*, 1.29 to 18.67% for the host, and less than 0.01% for *Archaea* (Fig. 2a). For both approaches, a substantial proportion of reads had no significant similarity to other sequences in the databases in GenBank (10.22 to 29.20%).

Overall, virion-enriched metagenomics and metatranscriptomics differed in the viruses detected as well as the expected counts (transcript abundance) as measured by RSEM analysis (Fig. 2c and d). Viruses from a wide range of viral groups were detected with metatranscriptomics, of which 49.87 to 97.51% had the closest hits to RNA viruses and 2.49 to 50.13% to DNA viruses. Conversely, for virion-enriched metagenomics, >95.54% of the virus-related sequences had the closest hits to DNA viruses, and <5% were identified as RNA viruses (Fig. 2b). Metatranscriptomics revealed high levels of viral diversity across all libraries, the most abundant viral groups detected being *Caudovirales*, *Luteo-Sobemo*, *Narna-Levi*, *Partiti-Picobirna*, *Picorna-Calici*, and *Tombus-Noda* (Fig. 2c). Conversely, virion-enriched metagenomics revealed relatively lower viral diversity across the same libraries; *Caudovirales* dominated the viral reads (69.89 to 99.49%), while viral groups identified at much lower abundances included *Microviridae*, *Circoviridae*, *Genomoviridae*, *Parvoviridae*, *Herpesviridae*, *Polyomaviridae*, and *Papillomaviridae* (Fig. 2c).

Vertebrate viruses detected by metatranscriptomics comprised 0 to 9.41% of the total viral reads. A large proportion of the viral reads belonged to either nonvertebrate eukaryotic viruses (45.08 to 97.51%), including plant viruses, insect viruses, and mycoviruses, or bacteriophage (2.48 to 48.91%) from the families *Siphoviridae*, *Podoviridae*, *Myoviridae*, and *Microviridae*. In the virion-enriched metagenomics data set, the percentage of vertebrate virus reads was also small (0.04 to 0.84%), while bacteriophage and other eukaryotic virus reads ranged between 79.17 and 99.91% and between 0.04 and 19.99%, respectively. Detailed information on all vertebrate viruses identified is presented in Table S1 in the supplemental material.

### Detection of viruses previously identified in other mammalian hosts (rabbit hemorrhagic disease virus and torovirus).

**FIG 3:**
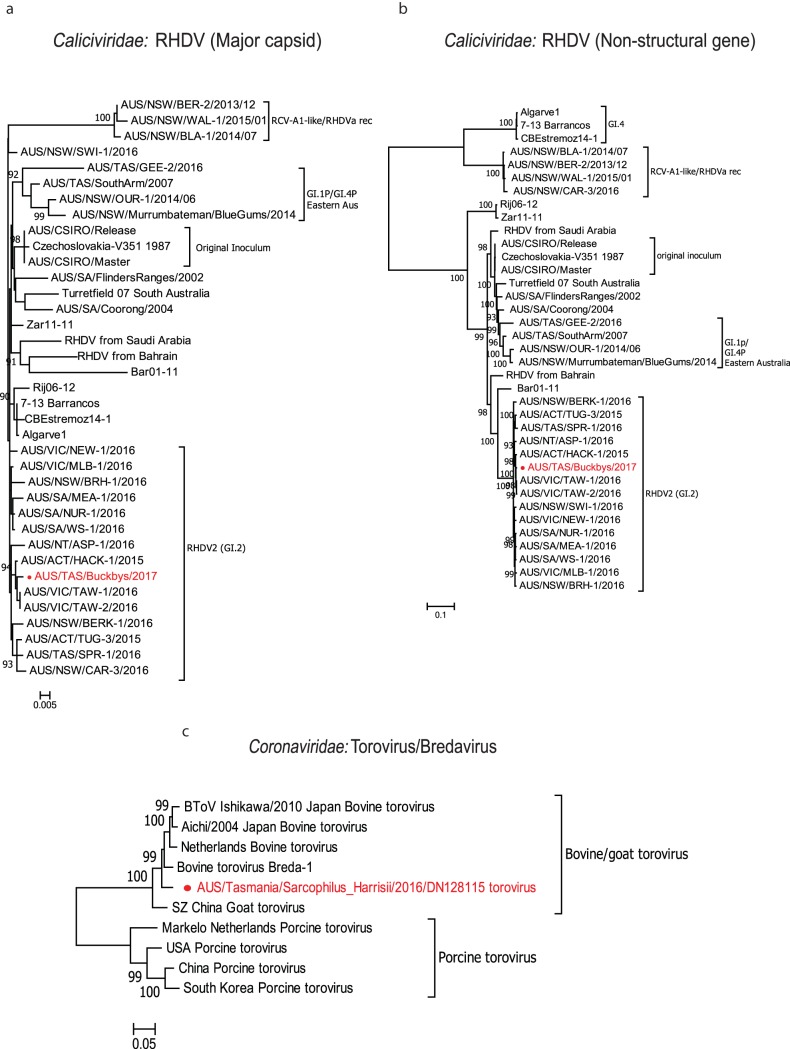
Maximum likelihood phylogenetic trees of viruses detected that were previously identified in other mammalian hosts. (a) Phylogenetic analysis of representative RHDV strains based on the 1,414-nucleotide (nt) sequence of major capsid protein. (b) Phylogenetic analysis of representative RHDV strains based on the 5,890-nt sequence of the nonstructural protein. (c) Phylogenetic analysis based on the 4,762-aa spike protein of the novel torovirus variant detected in this study with other previously identified toroviruses. All trees are mid-point rooted and scaled to either the number of amino acid substitutions or nucleotide substitutions per site based on the nature of alignment. Bootstrap values (>80%) are shown at the key nodes. The viral sequences detected in this study are shown in red in each tree.

Rabbit hemorrhagic disease virus (RHDV) is a calicivirus in the genus *Lagovirus* (22). RHDV is used as a biocontrol for rabbits in Australia and causes fatal hepatitis in European rabbits (Oryctolagus cuniculus) and some hare species (22). Using metatranscriptomics, we detected genomes with high nucleotide and amino acid similarity (>98%) to RHDV in one of the wild devil metatranscriptomic libraries (Buckbys Road [BR]), with genome coverage of 98.1%. Phylogenetic analysis based on the nucleotide sequences of the major capsid and nonstructural protein genes revealed that the RHDV detected in this study clustered with RHDV variant GI.2 (also called RHDV2) (Fig. 3a and b), which was first detected in Australia in May 2015 and has since become the dominant circulating variant nationwide. RHDV-specific reverse transcription-PCR (RT-PCR) and sequencing confirmed the presence of RHDV2 in four of the nine devil fecal samples from the BR metatranscriptomics pool. In addition, no rabbit-associated genes were detected during the initial sequence analysis. Additional PCR targeting a short fragment of rabbit mitochondrial DNA (mtDNA) (<300 bp) also did not detect any rabbit DNA in the original fecal samples from BR. Further RT-PCR and sequencing performed on the fecal RNA extractions from the remaining pools confirmed the presence of RHDV in 1 of 10 devils from wukalina/Mt William National Park (wMW), two of nine devils from zoo A, and one of nine from zoo B. One of the four additional RHDV-positive samples, from zoo A, contained rabbit mtDNA as confirmed by PCR.

We also identified the complete viral genome (28,463 bp) of a novel torovirus most closely related (96% nucleotide similarity) to bovine torovirus (Breda virus) in one of the metatranscriptomic libraries (wMW). We determined the full genome structure of the novel torovirus variant, including open reading frame (ORF) 1a and ORF 1b (encoding the two polyproteins pp1a and pp1ab), ORF 2 (encoding the spike protein [S]), ORF 3 (encoding the membrane protein [M]), ORF 4 (encoding the hemagglutinin-esterase protein [HE]), and ORF 5 (encoding the nucleocapsid protein [N]) (23). Based on the phylogenetic analysis of the spike protein amino acid sequence (4,762 amino acids [aa]), clustering of the novel torovirus variant with other toroviruses isolated from cattle in the United States, Japan, and Europe tentatively suggested a bovine origin, although this will need to be confirmed with wider sampling (Fig. 3c).

### Detection and characterization of novel marsupial-associated viruses. (i) Picornaviruses.

**FIG 4:**
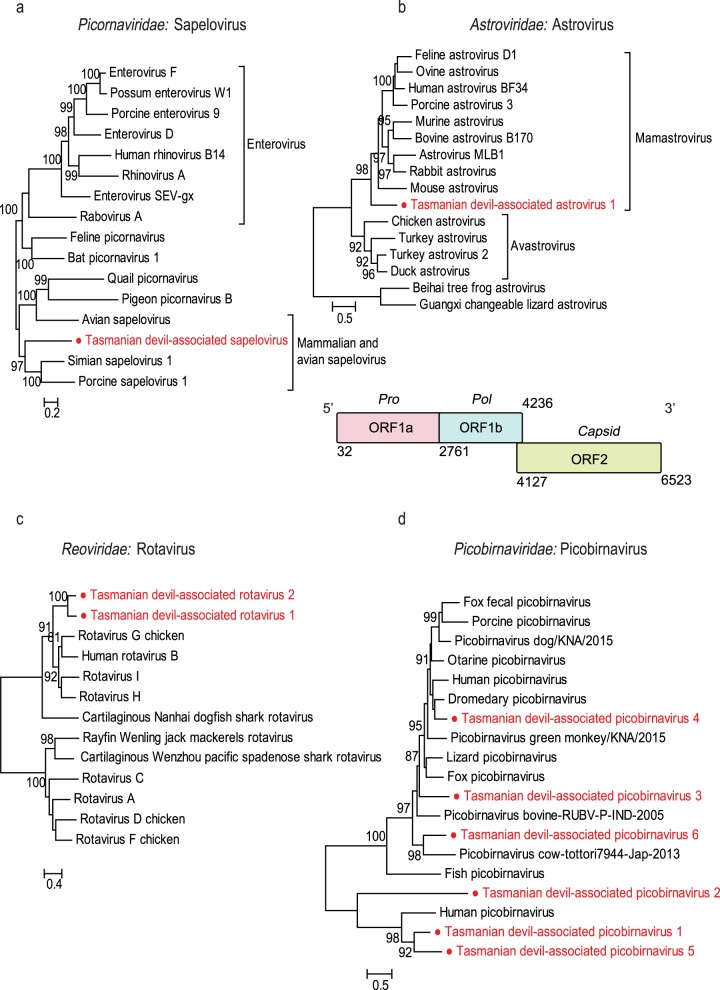
Phylogenetic analyses and genomic structures of the RNA viruses identified in the feces of Tasmanian devils. All phylogenetic analyses were performed based on the amino acid sequence of the RdRp. (a) Tasmanian devil-associated sapelovirus; (b) Tasmanian devil-associated astrovirus; (c) Tasmanian devil-associated rotaviruses 1 and 2; (d) Tasmanian devil-associated picobirnaviruses 1 to 6. For Tasmanian devil-associated astrovirus, for which the whole-genome sequence was obtained, the genomic structure is shown below the corresponding phylogenetic tree. Predicted ORFs of these genomes are labeled with information of the potential protein or protein domain they encode. All trees are mid-point rooted and scaled to the number amino acid substitutions per site. Bootstrap values (>80%) are shown at the key nodes. The newly discovered viruses are shown in red in each phylogenetic tree.

The complete genome (8,015 bp) of a novel virus in *Picornaviridae* was identified in one metatranscriptomic library (wMW). According to the International Committee on Taxonomy of Viruses (ICTV), members of a *Picornavirus* genus should share at least 40% amino acid sequence identity in the polyprotein region (23). The encoded 2,396-aa polyprotein of the virus detected in this study exhibited 45.5% amino acid similarity to simian sapelovirus, placing it in the genus *Sapelovirus*. We have provisionally named this newly identified virus Tasmanian devil-associated sapelovirus. Phylogenetic analysis based on the amino acid sequence of the RNA-dependent RNA polymerase (RdRp) domain showed that Tasmanian devil-associated sapelovirus formed a sister lineage to sapeloviruses identified from eutherian mammals (i.e., porcine and simian sapeloviruses) (Fig. 4a).

### (ii) Astroviruses.

Astrovirus-related sequences were detected in five of the six metatranscriptomic libraries. We identified one complete and one near-complete genome sequence with 81.4% pairwise nucleotide identity, involving two separate variants of a single astrovirus species, tentatively named Tasmanian devil-associated astrovirus 1 (24). The novel astrovirus identified here has a genome structure typical of other astroviruses, with three putative open reading frames (ORF 1a, ORF 1b, and ORF 2) encoding the protease, RdRp, and capsid, respectively. In addition, we found a ribosomal frameshift motif (AAAAAAC) within the ORF 1a/1b overlap region. Phylogenetic analysis based on the conserved RdRp domain showed that Tasmanian devil-associated astrovirus 1 formed a distinct cluster that is more closely related to astroviruses of mammalian hosts (mamastroviruses) than those of avian hosts (avastroviruses) (Fig. 4b).

### (iii) Rotaviruses.

Rotavirus sequences were identified in three metatranscriptomic libraries (Stony Head [SH], BR, and wMW). Among them, we identified two segments (3,481 bp and 3,479 bp) encoding rotavirus VP1 (i.e., RdRp). The two RdRp sequences shared >90% nucleotide identity with each other, indicative of two different variants from the same species. In addition, a contig encoding a partial rotavirus VP1 of 282 aa sharing 51% sequence similarity with rotavirus H was also detected in one library (wMW). We named the two viruses Tasmanian devil-associated rotavirus 1 and Tasmanian devil-associated rotavirus 2. The VP1 of Tasmanian devil-associated rotavirus 1 shared the highest amino acid similarity, ∼51%, with rotavirus G, while Tasmanian devil-associated rotavirus 2 shared the highest amino acid similarity, 44%, with rotavirus H. Phylogenetic analysis with other rotavirus species based on VP1 suggested that the two Tasmanian devil-associated rotaviruses form a distinct cluster most closely related to rotaviruses from avian and mammalian hosts (Fig. 4c).

### (iv) Picobirnaviruses.

We detected picobirnavirus sequences that encoded complete and partial viral RdRp (330 to 557 aa) in all four of the six metatranscriptomics libraries from wild devils. However, the picobirnavirus sequences detected in one library (Maria Island [MI]) were too short to be phylogenetically informative and were discarded in the phylogenetic analysis. The novel picobirnaviruses detected in this study are provisionally named Tasmanian devil-associated picobirnaviruses 1, 2, 3, 4, 5, and 6, with two separate variants in Tasmanian devil-associated picobirnaviruses 1 and 5. Phylogenetic analysis based on the RdRp domain of these novel picobirnaviruses showed that they are highly diverse and widely distributed across the phylogeny of this family (Fig. 4d).

### (v) Parvoviruses.

**FIG 5:**
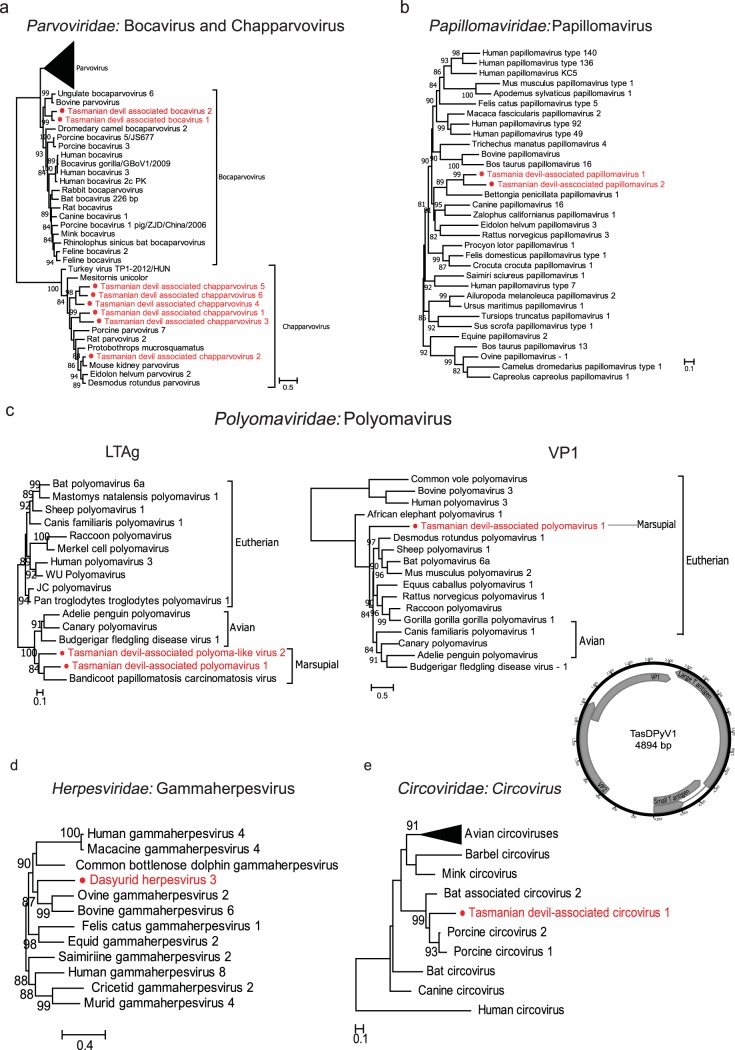
Phylogenetic analyses and genomic structures of the DNA viruses identified in the feces of Tasmanian devils. (a) Tasmanian devil-associated bocarviruses 1 and 2 of *Bocaparvovirus* and Tasmanian devil-associated chapparvoviruses 1 to 6 of *Chapparvoviru*s based on the amino acid sequences of the NS1 protein. (b) Tasmanian devil-associated papillomaviruses 1 and 2 based on the amino acids of the E1 protein. (c) Tasmanian devil-associated polyomavirus 1 and polyoma-like virus 2 based on the amino acid sequences of the LTAg and VP1 proteins. For Tasmanian devil-associated polyomavirus 1, the whole-genome sequence was obtained and the genomic structure is shown below the VP1 tree. Predicted ORFs of these genomes are labeled with information of the potential protein or protein domain they encode. (d) Dasyurid herpesvirus 3 based on the amino acid sequence of the DNA polymerase. (e) Tasmanian devil-associated circovirus 1 based on the amino acid sequence of the replicase protein. All trees are mid-point rooted and scaled to the number of amino acid substitutions per site. Bootstrap values (>80%) are shown at the key nodes. The newly discovered viruses are shown in red.

We identified eight new members of the vertebrate-associated subfamily *Parvovirinae*, including two from the genus *Bocaparvovirus* and six from the recently determined genus *Chapparvovirus*. We recovered partial and near-complete protein sequences sharing ∼50% identity to California sea lion bocavirus and porcine bocavirus, respectively. Two bocavirus-related sequences detected in this study shared >97% amino acid sequence similarity, indicating two separate variants of the same species, provisionally named Tasmanian devil-associated bocavirus 1. A third bocavirus-related sequence was also identified in virion-enriched metagenomics library BR, sharing 71.13% amino acid similarity with Tasmanian devil-associated bocavirus 1 and provisionally named Tasmanian devil-associated bocavirus 2. The six new chapparvoviruses identified in this study have been provisionally named Tasmanian devil-associated chapparvoviruses 1 to 6, which shared less than 70% amino acid identity among themselves. Phylogenetic analysis with representative viruses from the *Parvoviridae* family confirmed the clustering of the Tasmanian devil-associated bocaviruses within the diversity of mammalian bocaviruses, although the branching order involving them and other bocaviruses remains unresolved. The chapparvoviruses identified in this study clustered closely with other chapparvoviruses, including a recently described parvovirus associated with kidney diseases in mice (25) (Fig. 5a).

### (vi) Papillomaviruses.

Fragmented genomes of two novel species of papillomavirus were identified in one virion-enriched metagenomics library (MI), among which we retrieved two longer fragments (1,225 and 1,335 bp), both of which encode partial E1 protein, an ATP-dependent DNA helicase required for viral replication (26). The fragments share 64% similarity, suggesting two distinct papillomavirus species, tentatively named Tasmanian devil-associated papillomaviruses 1 and 2. Phylogenetic analysis based on the E1 protein showed that they form a distinct cluster with Bettongia penicillata papillomavirus type 1 (BpPV1) isolated from the woylie, a small marsupial species (Fig. 5b and Fig. S3). While the marsupial papillomaviruses viruses are clustered together in the phylogenetic tree, their relationship with viruses identified from eutherian mammals remains unresolved.

### (vii) Polyomaviruses.

Two novel polyomaviruses were detected in three virion-enriched metagenomics libraries (MI, wMW, and zoo B). We recovered the complete circular genome of 4,894 bp of the tentatively named Tasmanian devil-associated polyomavirus 1 and a partial gene sequence (2,251 bp) of the large T antigen (LTAg) protein for the second polyoma-like virus, tentatively named Tasmanian devil-associated polyoma-like virus 2 (Fig. 5c). Phylogenetic analyses revealed strikingly different evolutionary histories for the structural and nonstructural parts of the genome (Fig. 5c and Fig. S4 and S5), indicative of recombination (27). In the LTAg phylogeny, Tasmanian devil-associated polyomavirus 1 and Tasmanian devil-associated polyoma-like virus 2 formed a distinct lineage with another marsupial virus, bandicoot papillomatosis carcinomatosis virus type 2 (BPCV-2), which, in turn, clustered with polyomaviruses of avian hosts (Fig. 5c and Fig. S5). In contrast, in the VP1 phylogeny (Fig. S4), the marsupial virus showed no close relationship with the avian viruses. Interestingly, the bandicoot papillomatosis carcinomatosis viruses (BPCV-1 and -2) showed a close relationship to Tasmanian devil-associated polyomavirus 1 only in the LTAg region and not the VP1 region.

### (viii) Herpesviruses.

In one captive virion-enriched metagenomics library (zoo A), we identified 70 contigs matching different regions of a novel herpesvirus genome, provisionally named dasyurid herpesvirus 3 (DaHV-3), which totaled 62,821 bp in length and included partial gene sequences of the DNA polymerase (575 aa), major DNA binding protein (465 aa), helicase (396 aa), glycoproteins M (378 aa) and H (427 aa), and major capsid protein (358 aa), among others. On phylogenetic analysis based on these nonstructural and structural proteins, DaHV-3 clustered with other gammaherpesviruses (*Gammaherpesvirinae*) (Fig. S1 and S2). Further phylogenetic analysis based on the DNA polymerase showed that DaHV-3 forms a distinct lineage most closely related to bovine gammaherpesvirus 6 and ovine gammaherpesvirus 2 (Fig. 5d). The previously characterized dasyurid herpesvirus 2 (DaHV-2) isolated from devils (12) could not be included in the phylogenetic analysis because no sequences were available from the same genomic regions. A BLASTx search of the DNA polymerase showed that DaHV-3 exhibited the greatest amino acid similarity (93.3%) with macropodid herpesvirus 3 (MaHV-3) isolated previously from an Eastern gray Kangaroo (12), whose DNA polymerase amino acid sequence was also too short (<50% of the other representative herpesviruses) to be included in the phylogenetic analysis.

### (ix) Circoviruses.

We identified circovirus-related sequences and recovered the partial replicase gene sequences (899 bp) in one of the wild devil virion-enriched metagenomes (SH) and tentatively named it Tasmanian devil-associated circovirus. Phylogenetic analysis based on the Rep proteins with representative strains in the *Circovirus* genus suggested that Tasmanian devil-associated circovirus is clustered with circoviruses previously isolated from bats and pigs, sharing the highest sequence identity (62%) with a bat circovirus (GenBank accession number AIF76281) (Fig. 5e).

### Other viruses: plant and insect viruses and bacteriophage.

In both the virion-enriched metagenomics and metatranscriptomics analyses, large proportions of viral reads could be attributed to viruses that infect plants, insects, and bacteria, indicating ingestion of arthropods and/or environmental contamination of feces. Bacteriophage sequences from *Caudovirale*s were detected in all libraries, comprising >90% of all virion-enriched metagenomic viral reads and up to 48.91% of the metatranscriptomic viral reads. Sequences related to newly identified arthropod viruses, such as Wuhan fly virus and Wuhan mosquito virus, were also detected. Most of the insect viruses detected belong to the *Bunyavirales*, the *Mononegavirales*, and the *Chuviridae*, as well as the DNA virus subfamily *Densovirinae* (*Parvoviridae*). Sequences related to plant and fungal viruses were observed in all libraries, including sobemoviruses, tombusviruses, and mitoviruses.

### Comparison between devil populations.

Within-library viral diversity as characterized by our metatranscriptomics approach was significantly different between captive and wild populations (*P* < 0.05). In general, captive populations had lower diversity in their fecal viromes than wild populations.

**FIG 6:**
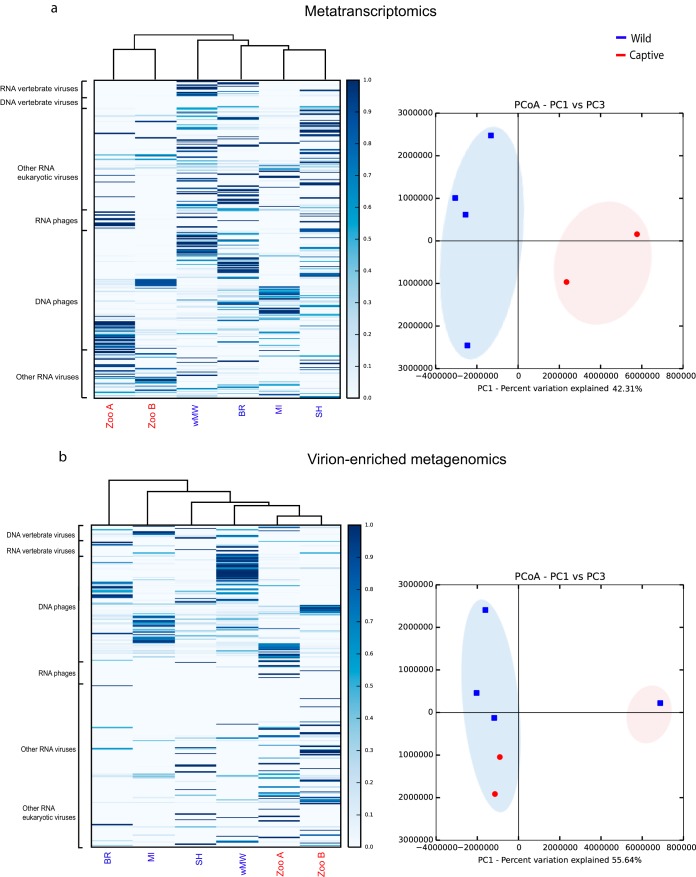
Heat maps (left) showing the hierarchical clustering and percentage of reads from each library mapping to various viral groups and principal-coordinate analysis (PCoA) plots (right) showing the similarity relations among libraries based on Euclidean distances as seen in metatranscriptomics (a) and virion-enriched metagenomics (b).

Metatranscriptomics analysis of MI devils displayed a level of overall viral diversity similar to those of both captive populations (zoo A and zoo B) and lower than that found in the other wild populations (Fig. 6a). Conversely, alpha diversity determined from virion-enriched metagenomics data did not differ significantly between libraries (Fig. 6b). Cluster analysis indicated that in metatranscriptomics, the wild and captive devils fell into two distinct clusters, while in virion-enriched metagenomics, BR formed its own cluster and the remaining populations formed a second cluster (Fig. 6).

## DISCUSSION

Both virion-enriched metagenomics and metatranscriptomics identified a wide diversity of viruses in the feces of Tasmanian devils, including vertebrate viruses, bacteriophages, and other eukaryotic viruses. Overall, we detected sequences related to 26 vertebrate viruses, including 24 novel marsupial-related viruses, including a sapelovirus, an astrovirus, rotaviruses, picobirnaviruses, bocaviruses, chapparvoviruses, papillomaviruses, polyomaviruses, and a gammaherpesvirus, as well as two known mammalian viruses, RHDV2 and torovirus.

However, there were also marked differences between the virion-enriched metagenomics and metatranscriptomics approaches. In general, virion-enriched metagenomics largely detected DNA viruses, while metatranscriptomics detected both DNA and RNA viruses, although the DNA viruses detected were limited to those with relatively high abundance (Fig. 2d). A high abundance level is often indicative of an active viral infection, during which DNA viruses are transcribed into RNA intermediates detected readily by RNA sequencing (28). Conversely, RNA viruses identified in metatranscriptomics were rarely detected in virion-enriched metagenomics, even if they were highly abundant based on the RSEM estimated counts.

The use of virion enrichment and sequence-independent amplification in our metagenomics approach increased the number of viral reads in each library. However, the viral compositions of all six virion-enriched metagenomes were highly skewed toward DNA viruses, particularly bacteriophage from the order *Caudovirales*. Similarly, in a previous study comparing various enrichment methods, bacteriophage accounted for >80% of all reads in all of the enrichment methods tested but <5% when no enrichment steps were incorporated (29). Despite being able to substantially increase the total number of viral reads in the metagenomes, sequence-independent amplification is bias prone, resulting in fewer viruses detected and lower genome coverage due to preferential amplification of certain sequences (30–32). However, the overrepresentation of bacteriophage here may partly be attributed to the fact that they make up the bulk of the gut virobiota, which is dominated by bacteria (33, 34). Regardless of its known bias (29, 30), virion-enriched metagenomics still holds merit for use in virome characterization due to its ability to identify low-abundance DNA viruses, which is especially relevant for dormant or nonactive viruses.

In contrast to virion-enriched metagenomics, metatranscriptomics is nonvirus specific and reveals the entire transcriptome within a sample (20, 35). Since there is no virion enrichment and less sample processing, the likelihood of biased detection is plausibly reduced in metatranscriptomics. In this study, the proportion of viral reads sequenced by metatranscriptomics was <2% per library, but the numbers of viral groups detected were significantly higher than those detected in virion-enriched metagenomics, which included both RNA and DNA viruses. Importantly, then, our results show that the taxonomic compositions of viral communities as revealed by virion-enriched metagenomics and metatranscriptomics were not interchangeable and neither approach was able to detect all viruses present. However, these two approaches were complementary, and an integrated approach using both virion-enriched metagenomics and metatranscriptomics will be a powerful tool for obtaining a complete overview of both the taxonomic and functional profiles of viral communities in a sample.

Ecological analysis of virome composition and diversity revealed significant differences between captive and wild devil populations, especially using metatranscriptomics. Both captive populations displayed lower levels of viral diversity than the wild populations. This loss of diversity is consistent with changes previously observed in the gut bacteriome, where captive devils also exhibited lower bacterial diversity compared to wild devils (11). Changes in lifestyle and diet that occur in captivity likely impact the virome, which is similarly dynamic. Interestingly, Maria Island devils had viromes more similar to those of the captive populations. Maria Island, a 115-km^2^ island off the east coast of Tasmania (Fig. 1), is home to approximately 100 free-ranging devils. Two factors may have contributed to the lower viral diversity observed on Maria Island. First, due to its isolation from mainland Tasmania, animal movements or immigrations are limited to only marine or bird species, and nonendemic species diversity is lower. Thus, there may be limited introduction of viruses to the island. Second, captive-born devils may be more likely to have a “captive-type” virome, that is, lower viral diversity than wild devils. Indeed, some devils included in this study are captive-born animals recently translocated to Maria Island. As samples were pooled prior to sequencing, it was not possible to distinguish between viromes of captive-born and wild-born devils.

While some of the viruses identified in this study come from families that include important pathogens, their pathogenic potential in devils is unclear. It is also important to note that some of these viruses may in fact be dietary viruses, with no active replication in devils, or may occur naturally as part of the normal gut flora of Tasmanian devils. For example, in some areas of Tasmania, rabbits are common and likely ingested by wild devils, which might explain the presence of RHDV2 in wild devils. They are also regularly fed to devils in captivity. Feeding records provided by the two zoos in this study confirmed the feeding of rabbits to some of the sampled devils around the time of fecal collection. While we did not detect rabbit genes by metatranscriptomics, virion-enriched metagenomics, or PCR in any of the wild devil samples that tested positive for RHDV, we did detect rabbit mtDNA in the feces of one captive devil from zoo A that tested positive for RHDV by PCR. Targeted investigations such as PCR of blood or internal tissues (i.e., liver), *in situ* hybridization, and serological assays are required to determine whether these viruses can actively replicate and cause disease in devils or are simply gut contaminants. Nevertheless, exposure to host-adapted viruses could pose significant health threats, especially for devils that are immunocompromised due to old age, DFTD, or other concurrent diseases (36, 37). Furthermore, even commensal or latent viral infections can be exacerbated or reactivated in immunocompromised hosts (38, 39).

Characterization of the gut virome in healthy devils in this study provides a fundamental baseline for future investigations of diseased animals. Tasmanian devils have low genetic diversity across their genomes and at functionally important loci such as the major histocompatibility complex (MHC) (40–42). This renders them particularly vulnerable to environmental changes, including the emergence of new pathogens, as seen in other species with low genetic diversity (43). For instance, in cheetahs, a coronavirus-associated feline infectious peritonitis outbreak causing mass mortality in a captive breeding colony was linked to the species’ extreme genetic monomorphism, particularly at the MHC (44).

Phylogenetic analyses of the newly identified viruses, including divergent members of their respective viral families, has provided insights into the evolutionary history of marsupial-associated viruses relative to viruses of eutherian mammals and other host taxa. Generally, long-term relationships between viruses and hosts are expected for mammalian viruses (13). Strong evidence for this lies in the observation that devil viruses are usually clustered with other marsupial viruses, as a marsupial-specific lineage that is distinct from the eutherian viruses, as observed in herpesvirus, papillomavirus (45, 46), and polyomavirus (Fig. S1 to S5). Furthermore, in several cases the branching order of viruses broadly reflects that of their hosts such that a general codivergence can be inferred. For instance, in the phylogenies of *Picornaviridae* and *Astroviridae*, the Tasmanian devil-associated (marsupial) viruses formed a sister clade to eutherian viruses, which, in turn, are sister to avian viruses, consistent with the evolutionary history of the host. Although such a relationship is not observed in every virus phylogeny, a deep divergence between eutherian and marsupial viruses is typical of our data set. This observation indicates that the timescale of virus evolution is very likely to reflect that of the hosts (13).

The gut virome is increasingly recognized as an integral component of the gut microbiome, and studies of the devil virome will continue to shine light on the biology and health of this iconic endangered species. For example, bacteriophage, which appear to dominate the devil’s fecal virome, can contribute to host health by maintaining the diversity and structure of the gut bacteriome through direct interactions with the bacterial communities. While the functions of bacteriophage on devil health remain to be determined, future studies will be able to exploit the extensive microbiomic data that are now available to answer important questions about host-microbe relationship between devils and their microbiome (11).

In sum, our identification of a broad array of vertebrate- and marsupial-specific viruses in devils provides potential candidate viruses for future disease surveillance as part of the broader conservation management of devils once the pathogenic potential of these viruses has been elucidated.

## MATERIALS AND METHODS

### Sample collection.

Fecal samples were collected from healthy wild Tasmanian devils between September 2016 and June 2017 from four locations in Tasmania (Fig. 1)—Stony Head (SH), Buckbys Road (BR), Maria Island (MI), and wukalina/Mt William National Park (wMW)—and from captive devils at two Australian mainland zoos in June and July 2017 (zoo A and zoo B). Devils were trapped overnight during routine monitoring by Save the Tasmanian Devil Program staff (47). Fresh fecal samples were collected directly from either the animal or the traps or restraint bags during processing of animals. All samples were stored in liquid nitrogen or a portable −80°C freezer (Stirling Ultracold, Global Cooling Inc.) immediately after collection. After arriving at the laboratory, samples were separated into two aliquots to be used in the extraction of total RNA for metatranscriptomics and enrichment of virions for virion-enriched metagenomics.

### Metatranscriptomics: total RNA extraction, library preparation, and sequencing.

Samples were disrupted and homogenized in 600 μl of lysis buffer with 1.44-mm ceramic beads using a Bead Ruptor homogenizer (Omni International) at 5 m·s^−1^ for 5 min. Total RNA was isolated using the Qiagen RNeasy Plus minikit following the manufacturer’s instructions. Extracted RNAs were pooled based on their source locations at equal mass amounts, with each pool containing 6 to 10 samples. Prior to library preparation, RNA pools were depleted of host and bacteria rRNA using a Ribo-Zero-Gold (epidemiology) kit (Illumina). Use of rRNA depletion instead of poly(A) enrichment ensures the retention of RNA with and without poly(A) tails. Sequencing libraries were constructed using a TruSeq total RNA library preparation kit (Illumina), and paired-end (75 bp) sequencing of each library was performed on a NextSeq500 HO platform (Illumina) at the Ramaciotti Centre for Genomics (Sydney, Australia).

### Virion-enriched metagenomics. (i) Virion enrichment and nucleic acid extraction.

A second aliquot from each fecal sample was processed for the virion-enriched metagenomics approach, as described previously, with minor modifications (17).

Fecal suspensions (10%) were homogenized for 1 min using the Bead Ruptor homogenizer (Omni International) at 5 m·s^−1^ and centrifuged at 15,000 × *g* for 3 min (Hitachi centrifuge, type CT15E; T15A62 fixed-angle rotor). Resulting supernatants were filtered through 0.45-μm membrane filters (Corning), and filtrates were treated with a cocktail of nucleases at 37°C for 2 h. Viral DNA and RNA were then simultaneously extracted using the QIAamp viral RNA minikit (Qiagen) (17).

### (ii) Random amplification.

Extracted viral nucleic acids were pooled as for the total RNA preparations for metatranscriptomics described above. Pooled extractions were subjected to first- and second-strand synthesis and random PCR amplification for 22 cycles using a complete whole-transcriptome amplification (WTA2) kit (Sigma-Aldrich) (17). WTA2 PCR products were then purified using Agencourt AMPure XP beads (Beckman Coulter) prior to library preparation and sequencing.

### (iii) Library preparation and Illumina sequencing.

Sequencing libraries were constructed using the Nextera XT DNA library preparation kit (Illumina) according to the manufacturer’s instructions, with modifications as described in reference 17. Paired-end (100 bp) sequencing of each library was performed on a Hiseq2500 platform (Illumina) at the Ramaciotti Centre for Genomics.

### Assembly and annotation.

Sequencing reads were demultiplexed and quality trimmed with Trimmomatic (48) and assembled *de novo* using Trinity (49). Resulting contigs were compared against the nonredundant nucleotide and protein databases on GenBank using BLASTN and BLASTX, respectively, with an E value cutoff at 1E−5. BLASTX searching was also conducted against a bespoke database containing all viral RNA-dependent RNA polymerase (RdRp) protein reference sequences downloaded from GenBank. Taxonomic information at the domain level (i.e., *Eukarya*, *Bacteria*, or *Archaea*), as well as for viruses, was assigned based first on the BLASTN results and then on the BLASTX results. Potential virus-related sequences were further categorized into families and orders based on their genetic similarity to their closest relatives and/or their phylogenetic positions. Similarly, assignment of viruses to the broader groups of their hosts (i.e., *Eukarya*, *Bacteria*, or *Archaea*) was based on their phylogenetic relationship to viruses with reliable host information obtained either using experimental or phylogenetic approaches. The genetic identity cutoff for host assignment was 40% based on the most conserved proteins, such as RdRp or DNA polymerases (50). The threshold was set based on the intrafamily diversity of most vertebrate-specific virus families/genera (13). The assignment of vertebrate host was based on phylogenetic analyses, in which a potential devil-associated virus is expected to either cluster within, or form a sister group to, an existing mammalian virus group.

To compare the abundances of transcripts/contigs, we calculated the percentage of total reads in each library. The abundance of host transcripts/contigs was estimated by mapping reads against the Tasmanian devil genome using Bowtie2 (51), whereas those of other organisms, namely, viruses, bacteria, archaea, and nonhost eukaryotes, were estimated using the RSEM approach (52) implemented in Trinity.

For each virus, the genome sequence was further extended by merging related contigs from the same or different pools. Gaps in the genome were filled either by aligning reads to contigs using Bowtie2 (51) or by RT-PCR and Sanger sequencing. Putative ORFs in viral genomes were predicted by the Geneious 8.1 software (53) and annotated based on similarity to previous published virus genomes.

### Phylogenetic analysis.

Nucleotide sequences of complete or partial genomes and amino acid sequences from the conserved domain (e.g., RdRp) of the newly characterized viral sequences were aligned with those of reference viruses representative of the diversity of the corresponding virus family or species. Alignment was performed using the E-INS-I algorithm implemented in MAFFT (version 7) (54). The quality of the alignments was subsequently assessed, and all ambiguously aligned regions were removed using TrimAl (version 1.2) (55). Phylogenetic trees of aligned amino acid (all data sets with the exception of RHDV) or nucleotide (RHDV) sequences were then inferred using the maximum likelihood method implemented in PhyML (version 3.0) (56), utilizing the best-fit substitution model and the Subtree Pruning and Regrafting (SPR) branch-swapping algorithm.

### Analyses of virome ecology.

Viral-abundance tables (Table S2 and Table S3) were generated based on complete or near-complete viral contigs and the percentage of reads mapped to them using Bowtie 2 (51) in each library. QIIME (version 1.9) (57) was used to perform ecological and statistical analysis to compare viromes of different populations. Within-library virotype richness (alpha diversity measured using the *Chao1* metric) and dissimilarity between libraries (beta diversity measured using the Euclidean metric) for both virion-enriched metagenomics and metatranscriptomics were calculated based on levels of viral abundance. Statistical significance of differences in alpha diversity was evaluated by the Monte Carlo method (999 permutations), with a null hypothesis that diversity is equal in all libraries with a significance threshold of α = 0.05. Levels of viral abundance were also used to produce heatmaps and dendrograms from hierarchical clustering. Principal-coordinate analysis (PCoA) was performed on the Euclidean distance matrix as calculated in QIIME, and additional cluster analysis was conducted using K-Means clustering in R (58).

### PCR confirmation and Sanger sequencing of RHDV.

Contigs with high similarity (>97%) to RHDV were detected in one of the metatranscriptomics libraries. To confirm the detection of RHDV, RT-PCR was performed on individual fecal RNA extractions using the Qiagen OneStep Ahead RT-PCR kit (Qiagen) and primers from a previously validated Australian RHDV strain-specific PCR (59) as well as two additional primer sets manually designed based on the current metatranscriptomics assembled contigs (Table 2). PCR products were separated on 1.5% agarose gels (Bio-Rad Laboratories) in 1× Tris-acetate EDTA and visualized using the SYBR Safe DNA gel stain (Life Technologies). Sanger sequencing of positive PCR products was performed at the Australian Genome Research Facility (Sydney, Australia). In addition, DNA was extracted from fecal samples using the Isolate fecal DNA kit (Bioline, London, UK), and the presence of rabbit DNA was tested using primers targeting a 110-bp region of the Oryctolagus cuniculus 12S mitochondrial rRNA gene (60). Rabbit DNA extracted from rabbit liver using the Bioline Isolate II genomic DNA kit (Bioline) was used as a positive control. Cleanup, primer trimming, and sequence analysis of Sanger data were performed using Geneious (53).

**TABLE 2:** List of primer sets used for PCR confirmation of RHDV2

Primer name	Sequence (5′→3′)	Amplicon size	Gene
Derived from assembled contigs			
BR_RHDV1_fwd	CTTGGCCAGTCTTTCTGGAG	168	RdRp
BR_RHDV1_Rev	ACAGGCAAACAGGTCCAAAC		
BR_RHDV3_fwd	ATCCCGCGCCGTACTGGGTTCCATTAG	405	RdRp
BR_RHDV3_Rev	GGGTCGGGTTTGGTGGGATCTGGAACA		
RHDV strain-specific multiplex (59)			
GI.1a-Aus_fwd	GCGTGGCATTGTGCGCAGCATC	562	RdRp
GI.1a-Aus_rev	TGTTGGTGATAAGCCATAATCGCG		
GI.1c_fwd	AGCAAGACTGTTGACTCAATTTCG	435	VP60
GI.1c_rev	AGGCCTGCACAGTCGTAACGTT		
GI.2_fwd	TTTCCCTGGAAGCAGTTCGTCA	336	VP60
GI.2_rev	TGTTGTCTGGTTTATGCCATTTGC		
GI.1a-K5_fwd	TTTATAGATGTATGCCCGCTCAAC	263	RdRp
GI.1a-K5_rev	CCGTTCGAGTTCCTTGCGGACG		

### Data availability.

Raw sequence reads generated in this study are available in NCBI SRA database under BioProject number PRJNA495667 (SRA: SRP165630). Viral genome sequences are available in GenBank (DNA viruses under accession numbers MK513523 to MK513543 and RNA viruses under accession numbers MK521912 to MK521927) and are available in the figshare repository (https://doi.org/10.6084/m9.figshare.7185146.v2).

## Supplementary Material

Supplemental file 1

Supplemental file 2

Supplemental file 3

Supplemental file 4
