# Supplementary material for: Fecal Viral Diversity of Captive and Wild Tasmanian Devils Characterized Using Virion-Enriched Metagenomics and Metatranscriptomics
Source: J Virol. 2019 May 15;93(11):e00205-19. doi: 10.1128/JVI.00205-19 (PMC6532096; doi:10.1128/JVI.00205-19)
Supplement: Supplemental file 4 [file JVI.00205-19-s0004.pdf]

Supplemental material

Fig. S1. Maximum likelihood phylogenetic tree of Dasyurid herpesvirus 3 in the context of representatives of the *Herpesviridae* family based on the amino acid sequences of glycoprotein M. The tree is mid-point rooted and scaled to the number of amino acid substitutions per site. Bootstrap values (>80%) are shown at the key nodes. The newly discovered Dasyurid herpesvirus 3 is shown in red.

Fig. S2. Maximum likelihood phylogenetic tree of Dasyurid herpesvirus 3 in the context of representatives of the *Herpesviridae* family based on the amino acid sequences of major capsid. The tree is mid-point rooted and scaled to the number of amino acid substitutions per site. Bootstrap values (>80%) are shown at the key nodes. The newly discovered DaHV-3 is shown in red.

Fig. S3. Maximum likelihood phylogenetic tree of Tasmanian devil-associated papillomavirus 1 and 2 in the context of representatives of the *Papillomaviridae* family based on the amino acid sequences of E1 protein. The tree is mid-point rooted and scaled to the number of amino acid substitutions per site. Bootstrap values (>80%) are shown at the key nodes. The 2 newly discovered papillomarviruses is shown in red.

Fig. S4. Maximum likelihood phylogenetic tree of Tasmanian devil-associated polyomavirus 1 in the context of representatives of the *Polyomarviridae* family based on the amino acid sequences of VP1. The tree is mid-point rooted and scaled to the number of amino acid substitutions per site. Bootstrap values (>80%) are shown at the key nodes. The newly discovered polyomavirus is shown in red.

Fig. S5. Maximum likelihood phylogenetic tree of Tasmanian devil-associated polyomavirus 1 and Tasmanian devil-associated polyoma-like virus 2 in the context of representatives of the *Polyomaviridae* family based on the amino acid sequences of the large T antigen. The tree is mid-point rooted and scaled to the number of amino acid substitutions per site. Bootstrap values (>80%) are shown at the key nodes. The 2 newly discovered viruses are shown in red.

Fig. S1

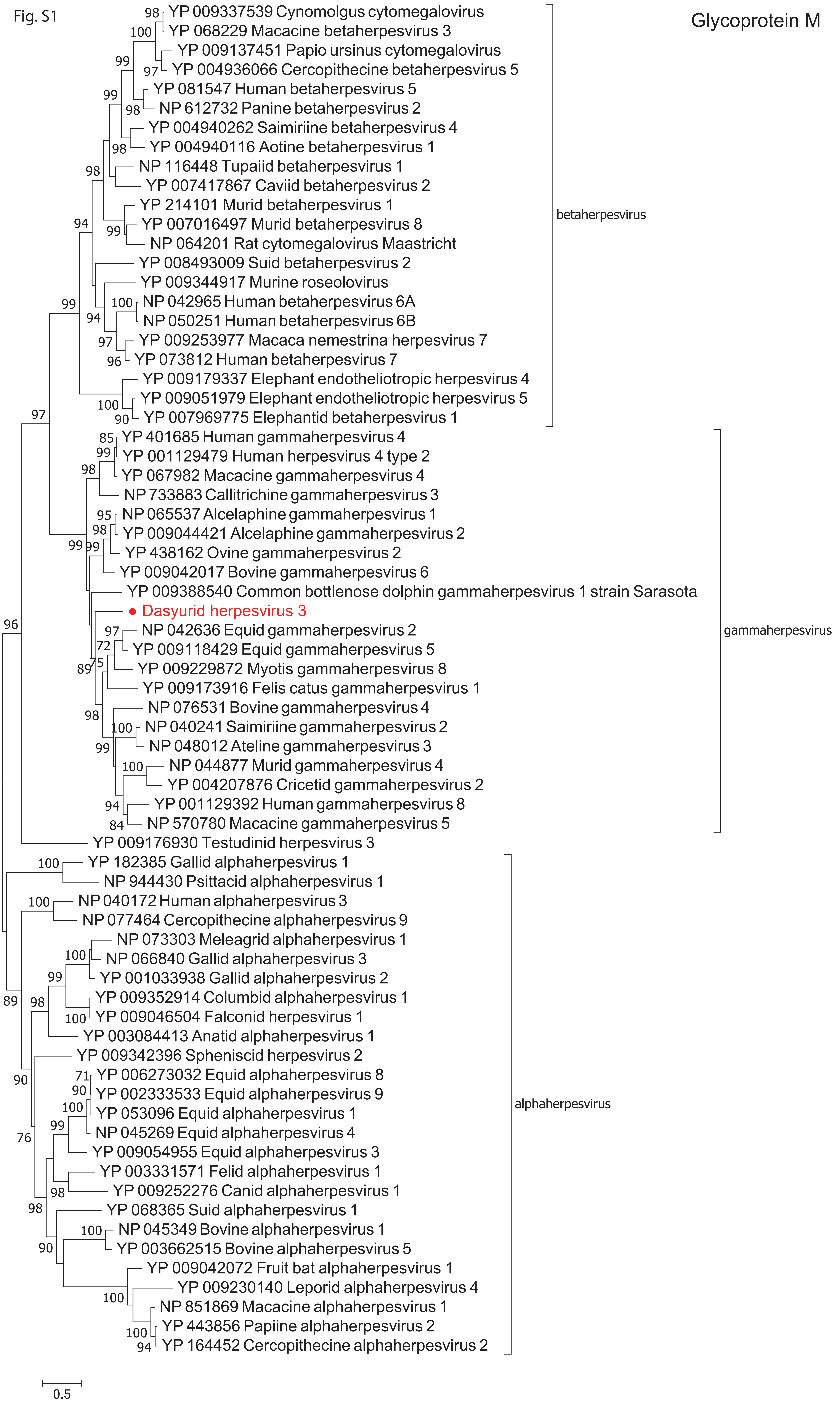
Glycoprotein M
YP 009337539 Cynomolgus cytomegalovirus
YP 068229 Macacine betaherpesvirus 3
YP 009137451 Papio ursinus cytomegalovirus
YP 004936066 Cercopithecine betaherpesvirus 5
YP 081547 Human betaherpesvirus 5
NP 612732 Panine betaherpesvirus 2
YP 004940262 Saimiriine betaherpesvirus 4
YP 004940116 Aotine betaherpesvirus 1
NP 116448 Tupaiid betaherpesvirus 1
YP 007417867 Caviid betaherpesvirus 2
YP 214101 Murid betaherpesvirus 1
YP 007016497 Murid betaherpesvirus 8
NP 064201 Rat cytomegalovirus Maastricht
YP 008493009 Suid betaherpesvirus 2
YP 009344917 Murine roseolovirus
NP 042965 Human betaherpesvirus 6A
NP 050251 Human betaherpesvirus 6B
YP 009253977 Macaca nemestrina herpesvirus 7
YP 073812 Human betaherpesvirus 7
YP 009179337 Elephant endotheliotropic herpesvirus 4
YP 009051979 Elephant endotheliotropic herpesvirus 5
YP 007969775 Elephantid betaherpesvirus 1
betaherpesvirus
YP 401685 Human gammaherpesvirus 4
YP 001129479 Human herpesvirus 4 type 2
YP 067982 Macacine gammaherpesvirus 4
NP 733883 Callitrichine gammaherpesvirus 3
NP 065537 Alcelaphine gammaherpesvirus 1
YP 009044421 Alcelaphine gammaherpesvirus 2
YP 438162 Ovine gammaherpesvirus 2
YP 009042017 Bovine gammaherpesvirus 6
YP 009388540 Common bottlenose dolphin gammaherpesvirus 1 strain Sarasota
Dasyurid herpesvirus 3
NP 042636 Equid gammaherpesvirus 2
YP 009118429 Equid gammaherpesvirus 5
YP 009229872 Myotis gammaherpesvirus 8
YP 009173916 Felis catus gammaherpesvirus 1
NP 076531 Bovine gammaherpesvirus 4
NP 040241 Saimiriine gammaherpesvirus 2
NP 048012 Ateline gammaherpesvirus 3
NP 044877 Murid gammaherpesvirus 4
YP 004207876 Cricetid gammaherpesvirus 2
YP 001129392 Human gammaherpesvirus 8
NP 570780 Macacine gammaherpesvirus 5
gammaherpesvirus
YP 009176930 Testudinid herpesvirus 3
YP 182385 Gallid alphaherpesvirus 1
NP 944430 Psittacid alphaherpesvirus 1
NP 040172 Human alphaherpesvirus 3
NP 077464 Cercopithecine alphaherpesvirus 9
NP 073303 Meleagrid alphaherpesvirus 1
NP 066840 Gallid alphaherpesvirus 3
YP 001033938 Gallid alphaherpesvirus 2
YP 009352914 Columbid alphaherpesvirus 1
YP 009046504 Falconid herpesvirus 1
YP 003084413 Anatid alphaherpesvirus 1
YP 009342396 Spheniscid herpesvirus 2
YP 006273032 Equid alphaherpesvirus 8
YP 002333533 Equid alphaherpesvirus 9
YP 053096 Equid alphaherpesvirus 1
NP 045269 Equid alphaherpesvirus 4
YP 009054955 Equid alphaherpesvirus 3
YP 003331571 Felid alphaherpesvirus 1
YP 009252276 Canid alphaherpesvirus 1
YP 068365 Suid alphaherpesvirus 1
NP 045349 Bovine alphaherpesvirus 1
YP 003662515 Bovine alphaherpesvirus 5
YP 009042072 Fruit bat alphaherpesvirus 1
YP 009230140 Leporid alphaherpesvirus 4
NP 851869 Macacine alphaherpesvirus 1
YP 443856 Papiine alphaherpesvirus 2
YP 164452 Cercopithecine alphaherpesvirus 2
alphaherpesvirus
0.5

Fig. S2

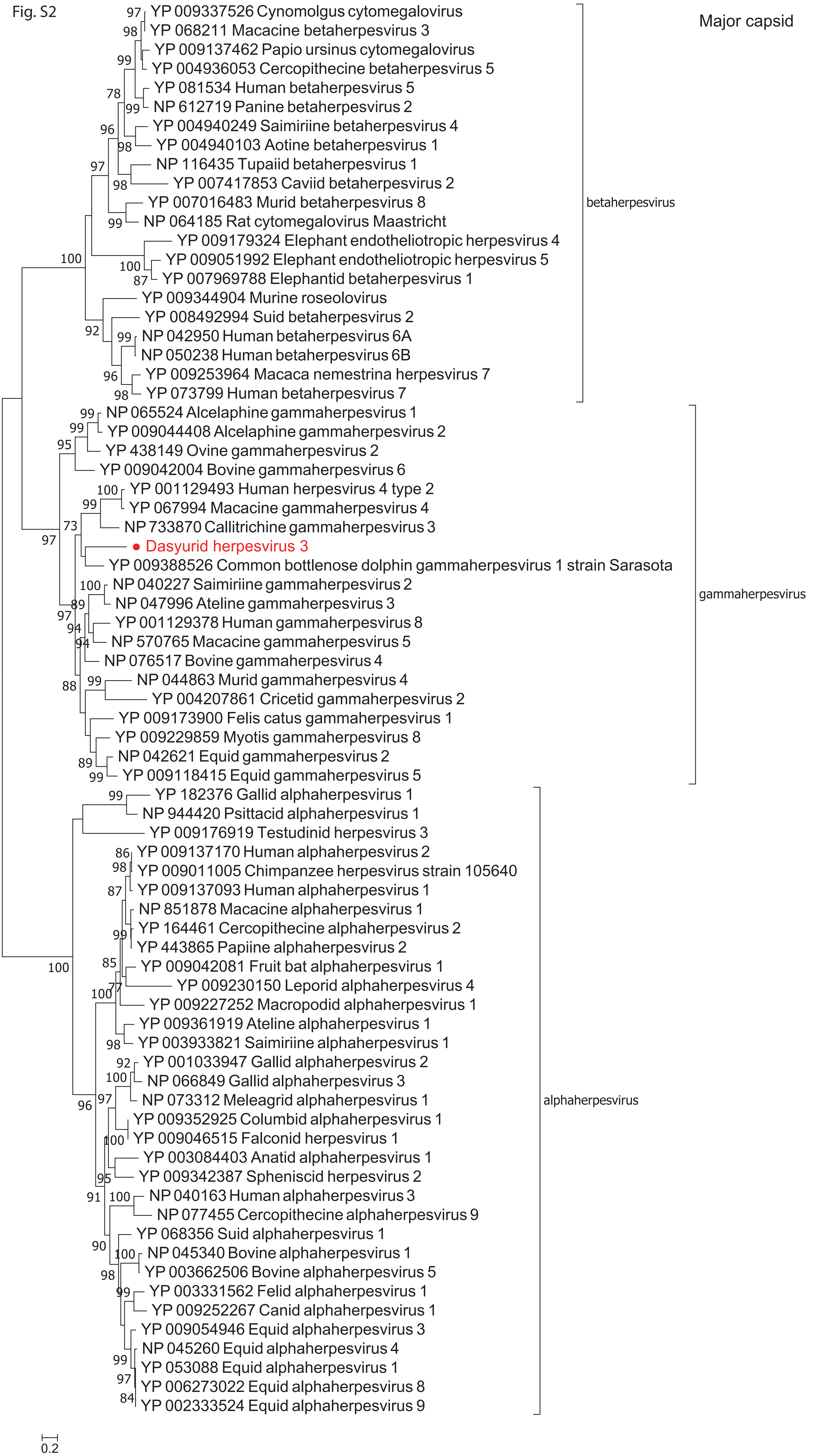
Major capsid
YP 009337526 Cynomolgus cytomegalovirus
YP 068211 Macacine betaherpesvirus 3
YP 009137462 Papio ursinus cytomegalovirus
YP 004936053 Cercopithecine betaherpesvirus 5
YP 081534 Human betaherpesvirus 5
NP 612719 Panine betaherpesvirus 2
YP 004940249 Saimiriine betaherpesvirus 4
YP 004940103 Aotine betaherpesvirus 1
NP 116435 Tupaiid betaherpesvirus 1
YP 007417853 Caviid betaherpesvirus 2
YP 007016483 Murid betaherpesvirus 8
NP 064185 Rat cytomegalovirus Maastricht
YP 009179324 Elephant endotheliotropic herpesvirus 4
YP 009051992 Elephant endotheliotropic herpesvirus 5
YP 007969788 Elephantid betaherpesvirus 1
YP 009344904 Murine roseolovirus
YP 008492994 Suid betaherpesvirus 2
NP 042950 Human betaherpesvirus 6A
NP 050238 Human betaherpesvirus 6B
YP 009253964 Macaca nemestrina herpesvirus 7
YP 073799 Human betaherpesvirus 7
betaherpesvirus
NP 065524 Alcelaphine gammaherpesvirus 1
YP 009044408 Alcelaphine gammaherpesvirus 2
YP 438149 Ovine gammaherpesvirus 2
YP 009042004 Bovine gammaherpesvirus 6
YP 001129493 Human herpesvirus 4 type 2
YP 067994 Macacine gammaherpesvirus 4
NP 733870 Callitrichine gammaherpesvirus 3
● Dasyurid herpesvirus 3
YP 009388526 Common bottlenose dolphin gammaherpesvirus 1 strain Sarasota
NP 040227 Saimiriine gammaherpesvirus 2
NP 047996 Ateline gammaherpesvirus 3
YP 001129378 Human gammaherpesvirus 8
NP 570765 Macacine gammaherpesvirus 5
NP 076517 Bovine gammaherpesvirus 4
NP 044863 Murid gammaherpesvirus 4
YP 004207861 Cricetid gammaherpesvirus 2
YP 009173900 Felis catus gammaherpesvirus 1
YP 009229859 Myotis gammaherpesvirus 8
NP 042621 Equid gammaherpesvirus 2
YP 009118415 Equid gammaherpesvirus 5
gammaherpesvirus
YP 182376 Gallid alphaherpesvirus 1
NP 944420 Psittacid alphaherpesvirus 1
YP 009176919 Testudinid herpesvirus 3
YP 009137170 Human alphaherpesvirus 2
YP 009011005 Chimpanzee herpesvirus strain 105640
YP 009137093 Human alphaherpesvirus 1
NP 851878 Macacine alphaherpesvirus 1
YP 164461 Cercopithecine alphaherpesvirus 2
YP 443865 Papiine alphaherpesvirus 2
YP 009042081 Fruit bat alphaherpesvirus 1
YP 009230150 Leporid alphaherpesvirus 4
YP 009227252 Macropodid alphaherpesvirus 1
YP 009361919 Ateline alphaherpesvirus 1
YP 003933821 Saimiriine alphaherpesvirus 1
YP 001033947 Gallid alphaherpesvirus 2
NP 066849 Gallid alphaherpesvirus 3
NP 073312 Meleagrid alphaherpesvirus 1
YP 009352925 Columbid alphaherpesvirus 1
YP 009046515 Falconid herpesvirus 1
YP 003084403 Anatid alphaherpesvirus 1
YP 009342387 Spheniscid herpesvirus 2
NP 040163 Human alphaherpesvirus 3
NP 077455 Cercopithecine alphaherpesvirus 9
YP 068356 Suid alphaherpesvirus 1
NP 045340 Bovine alphaherpesvirus 1
YP 003662506 Bovine alphaherpesvirus 5
YP 003331562 Felid alphaherpesvirus 1
YP 009252267 Canid alphaherpesvirus 1
YP 009054946 Equid alphaherpesvirus 3
NP 045260 Equid alphaherpesvirus 4
YP 053088 Equid alphaherpesvirus 1
YP 006273022 Equid alphaherpesvirus 8
YP 002333524 Equid alphaherpesvirus 9
alphaherpesvirus
0.2

Fig. S3

E1

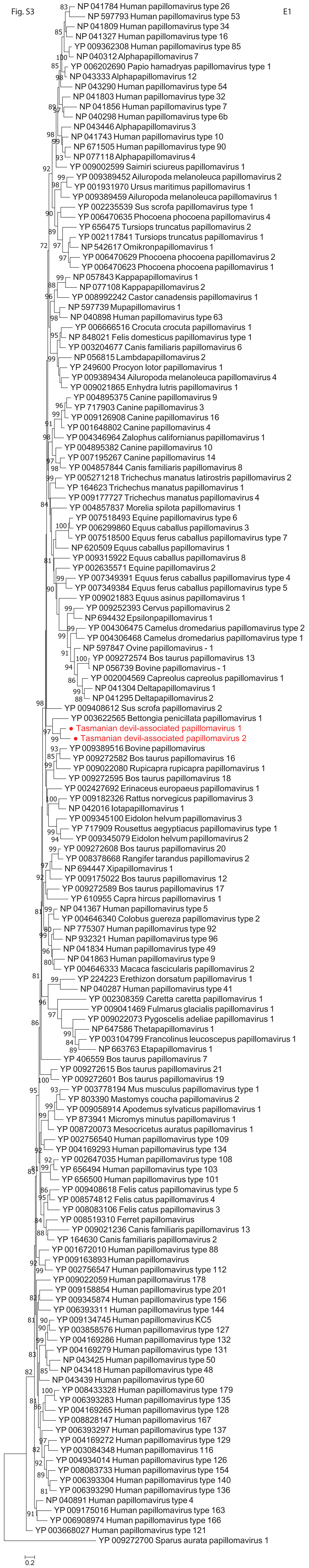

NP 041784 Human papillomavirus type 26
NP 597793 Human papillomavirus type 53
NP 041809 Human papillomavirus type 34
NP 041327 Human papillomavirus type 16
YP 009362308 Human papillomavirus type 85
NP 040312 Alphapapillomavirus 7
YP 006202690 Papio hamadryas papillomavirus type 1
NP 043333 Alphapapillomavirus 12
NP 043290 Human papillomavirus type 54
NP 041803 Human papillomavirus type 32
NP 041856 Human papillomavirus type 7
NP 040298 Human papillomavirus type 6b
NP 043446 Alphapapillomavirus 3
NP 041743 Human papillomavirus type 10
NP 671505 Human papillomavirus type 90
NP 077118 Alphapapillomavirus 4
YP 009002599 Saimiri sciureus papillomavirus 1
YP 009389452 Ailuropoda melanoleuca papillomavirus 2
YP 001931970 Ursus maritimus papillomavirus 1
YP 009389459 Ailuropoda melanoleuca papillomavirus 1
YP 002235539 Sus scrofa papillomavirus type 1
YP 006470635 Phocoena phocoena papillomavirus 4
YP 656475 Tursiops truncatus papillomavirus 2
YP 002117841 Tursiops truncatus papillomavirus 1
NP 542617 Omikronpapillomavirus 1
YP 006470629 Phocoena phocoena papillomavirus 2
YP 006470623 Phocoena phocoena papillomavirus 1
NP 057843 Kappapapillomavirus 1
NP 077108 Kappapapillomavirus 2
YP 008992242 Castor canadensis papillomavirus 1
NP 597739 Mupapillomavirus 1
NP 040898 Human papillomavirus type 63
YP 006666516 Crocuta crocuta papillomavirus 1
NP 848021 Felis domesticus papillomavirus type 1
YP 003204677 Canis familiaris papillomavirus 6
NP 056815 Lambdapapillomavirus 2
YP 249600 Procyon lotor papillomavirus 1
YP 009389434 Ailuropoda melanoleuca papillomavirus 4
YP 009021865 Enhydra lutris papillomavirus 1
YP 004895375 Canine papillomavirus 9
YP 717903 Canine papillomavirus 3
YP 009126908 Canine papillomavirus 16
YP 001648802 Canine papillomavirus 4
YP 004346964 Zalophus californianus papillomavirus 1
YP 004895382 Canine papillomavirus 10
YP 007195267 Canine papillomavirus 14
YP 004857844 Canis familiaris papillomavirus 8
YP 005271218 Trichechus manatus latirostris papillomavirus 2
YP 164623 Trichechus manatus papillomavirus 1
YP 009177727 Trichechus manatus papillomavirus 4
YP 004857837 Morelia spilota papillomavirus 1
YP 007518493 Equine papillomavirus type 6
YP 006299860 Equus caballus papillomavirus 3
YP 007518500 Equus ferus caballus papillomavirus type 7
NP 620509 Equus caballus papillomavirus 1
YP 009315922 Equus caballus papillomavirus 8
YP 002635571 Equine papillomavirus 2
YP 007349391 Equus ferus caballus papillomavirus type 4
YP 007349384 Equus ferus caballus papillomavirus type 5
YP 009021883 Equus asinus papillomavirus 1
YP 009252393 Cervus papillomavirus 2
NP 694432 Epsilonpapillomavirus 1
YP 004306475 Camelus dromedarius papillomavirus type 2
YP 004306468 Camelus dromedarius papillomavirus type 1
NP 597847 Ovine papillomavirus - 1
YP 009272574 Bos taurus papillomavirus 13
NP 056739 Bovine papillomavirus - 1
YP 002004569 Capreolus capreolus papillomavirus 1
NP 041304 Deltapapillomavirus 1
NP 041295 Deltapapillomavirus 2
YP 009408612 Sus scrofa papillomavirus 2
YP 003622565 Bettongia penicillata papillomavirus 1
Tasmanian devil-associated papillomavirus 1
Tasmanian devil-associated papillomavirus 2
YP 009389516 Bovine papillomavirus
YP 009272582 Bos taurus papillomavirus 16
YP 009022080 Rupicapra rupicapra papillomavirus 1
YP 009272595 Bos taurus papillomavirus 18
YP 002427692 Erinaceus europaeus papillomavirus 1
YP 009182326 Rattus norvegicus papillomavirus 3
NP 042016 Iotapapillomavirus 1
YP 009345100 Eidolon helvum papillomavirus 3
YP 717909 Rousettus aegyptiacus papillomavirus type 1
YP 009345079 Eidolon helvum papillomavirus 2
YP 009272608 Bos taurus papillomavirus 20
YP 008378668 Rangifer tarandus papillomavirus 2
NP 694447 Xipapillomavirus 1
YP 009175022 Bos taurus papillomavirus 12
YP 009272589 Bos taurus papillomavirus 17
YP 610955 Capra hircus papillomavirus 1
NP 041367 Human papillomavirus type 5
YP 004646340 Colobus guereza papillomavirus type 2
NP 775307 Human papillomavirus type 92
NP 932321 Human papillomavirus type 96
NP 041834 Human papillomavirus type 49
NP 041863 Human papillomavirus type 9
YP 004646333 Macaca fascicularis papillomavirus 2
YP 224223 Erethizon dorsatum papillomavirus 1
NP 040287 Human papillomavirus type 41
YP 002308359 Caretta caretta papillomavirus 1
YP 009041469 Fulmarus glacialis papillomavirus 1
YP 009022073 Pygoscelis adeliae papillomavirus 1
NP 647586 Thetapapillomavirus 1
YP 003104799 Francolinus leucoscepus papillomavirus 1
NP 663763 Etapapillomavirus 1
YP 406559 Bos taurus papillomavirus 7
YP 009272615 Bos taurus papillomavirus 21
YP 009272601 Bos taurus papillomavirus 19
YP 003778194 Mus musculus papillomavirus type 1
YP 803390 Mastomys coucha papillomavirus 2
YP 009058914 Apodemus sylvaticus papillomavirus 1
YP 873941 Micromys minutus papillomavirus 1
YP 008720073 Mesocricetus auratus papillomavirus 1
YP 002756540 Human papillomavirus type 109
YP 004169293 Human papillomavirus type 134
YP 002647035 Human papillomavirus type 108
YP 656494 Human papillomavirus type 103
YP 656500 Human papillomavirus type 101
YP 009408618 Felis catus papillomavirus type 5
YP 008574812 Felis catus papillomavirus 4
YP 008083106 Felis catus papillomavirus 3
YP 008519310 Ferret papillomavirus
YP 009021236 Canis familiaris papillomavirus 13
YP 164630 Canis familiaris papillomavirus 2
YP 001672010 Human papillomavirus type 88
YP 009163893 Human papillomavirus
YP 002756547 Human papillomavirus type 112
YP 009022059 Human papillomavirus 178
YP 009158854 Human papillomavirus type 201
YP 009345874 Human papillomavirus type 156
YP 006393311 Human papillomavirus type 144
YP 009134745 Human papillomavirus KC5
YP 003858576 Human papillomavirus type 127
YP 004169286 Human papillomavirus type 132
YP 004169279 Human papillomavirus type 131
NP 043425 Human papillomavirus type 50
NP 043418 Human papillomavirus type 48
NP 043439 Human papillomavirus type 60
YP 008433328 Human papillomavirus type 179
YP 006393283 Human papillomavirus type 135
YP 004169265 Human papillomavirus type 128
YP 008828147 Human papillomavirus 167
YP 006393297 Human papillomavirus type 137
YP 004169272 Human papillomavirus type 129
YP 003084348 Human papillomavirus 116
YP 004934014 Human papillomavirus type 126
YP 008083733 Human papillomavirus type 154
YP 006393304 Human papillomavirus type 140
YP 006393290 Human papillomavirus type 136
NP 040891 Human papillomavirus type 4
YP 009175016 Human papillomavirus type 163
YP 006908974 Human papillomavirus type 166
YP 003668027 Human papillomavirus type 121
YP 009272700 Sparus aurata papillomavirus 1
0.2

Fig. S4

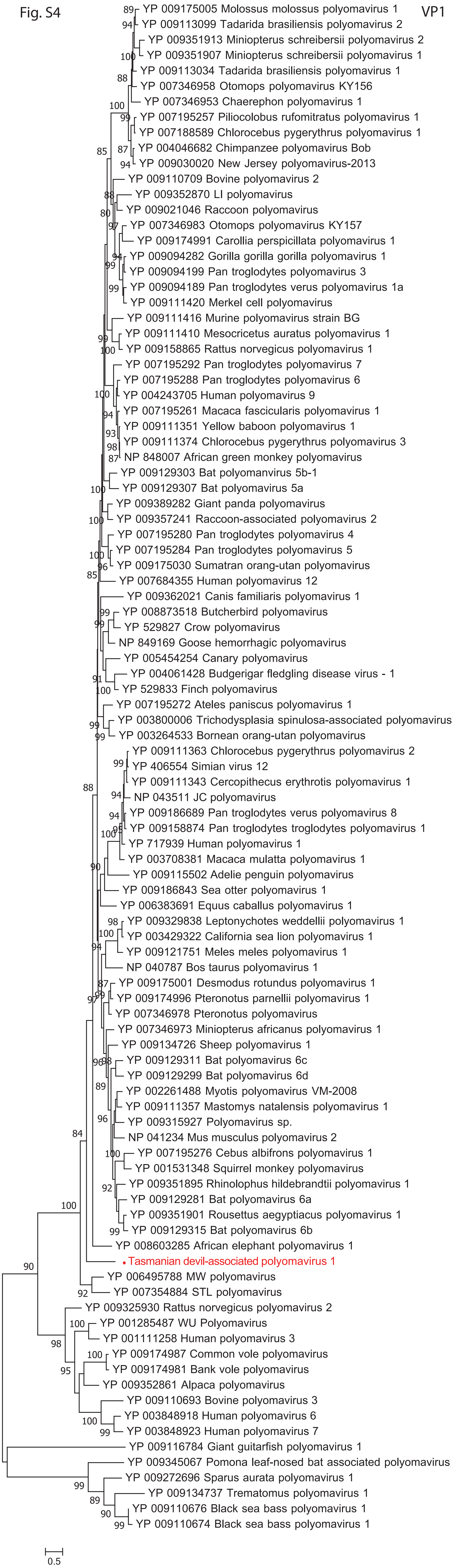
VP1
YP 009175005 Molossus molossus polyomavirus 1
YP 009113099 Tadarida brasiliensis polyomavirus 2
YP 009351913 Miniopterus schreibersii polyomavirus 2
YP 009351907 Miniopterus schreibersii polyomavirus 1
YP 009113034 Tadarida brasiliensis polyomavirus 1
YP 007346958 Otomops polyomavirus KY156
YP 007346953 Chaerephon polyomavirus 1
YP 007195257 Piliocolobus rufomitratus polyomavirus 1
YP 007188589 Chlorocebus pygerythrus polyomavirus 1
YP 004046682 Chimpanzee polyomavirus Bob
YP 009030020 New Jersey polyomavirus-2013
YP 009110709 Bovine polyomavirus 2
YP 009352870 LI polyomavirus
YP 009021046 Raccoon polyomavirus
YP 007346983 Otomops polyomavirus KY157
YP 009174991 Carollia perspicillata polyomavirus 1
YP 009094282 Gorilla gorilla gorilla polyomavirus 1
YP 009094199 Pan troglodytes polyomavirus 3
YP 009094189 Pan troglodytes verus polyomavirus 1a
YP 009111420 Merkel cell polyomavirus
YP 009111416 Murine polyomavirus strain BG
YP 009111410 Mesocricetus auratus polyomavirus 1
YP 009158865 Rattus norvegicus polyomavirus 1
YP 007195292 Pan troglodytes polyomavirus 7
YP 007195288 Pan troglodytes polyomavirus 6
YP 004243705 Human polyomavirus 9
YP 007195261 Macaca fascicularis polyomavirus 1
YP 009111351 Yellow baboon polyomavirus 1
YP 009111374 Chlorocebus pygerythrus polyomavirus 3
NP 848007 African green monkey polyomavirus
YP 009129303 Bat polyomanvirus 5b-1
YP 009129307 Bat polyomavirus 5a
YP 009389282 Giant panda polyomavirus
YP 009357241 Raccoon-associated polyomavirus 2
YP 007195280 Pan troglodytes polyomavirus 4
YP 007195284 Pan troglodytes polyomavirus 5
YP 009175030 Sumatran orang-utan polyomavirus
YP 007684355 Human polyomavirus 12
YP 009362021 Canis familiaris polyomavirus 1
YP 008873518 Butcherbird polyomavirus
YP 529827 Crow polyomavirus
NP 849169 Goose hemorrhagic polyomavirus
YP 005454254 Canary polyomavirus
YP 004061428 Budgerigar fledgling disease virus - 1
YP 529833 Finch polyomavirus
YP 007195272 Ateles paniscus polyomavirus 1
YP 003800006 Trichodysplasia spinulosa-associated polyomavirus
YP 003264533 Bornean orang-utan polyomavirus
YP 009111363 Chlorocebus pygerythrus polyomavirus 2
YP 406554 Simian virus 12
YP 009111343 Cercopithecus erythrotis polyomavirus 1
NP 043511 JC polyomavirus
YP 009186689 Pan troglodytes verus polyomavirus 8
YP 009158874 Pan troglodytes troglodytes polyomavirus 1
YP 717939 Human polyomavirus 1
YP 003708381 Macaca mulatta polyomavirus 1
YP 009115502 Adelie penguin polyomavirus
YP 009186843 Sea otter polyomavirus 1
YP 006383691 Equus caballus polyomavirus 1
YP 009329838 Leptonychotes weddellii polyomavirus 1
YP 003429322 California sea lion polyomavirus 1
YP 009121751 Meles meles polyomavirus 1
NP 040787 Bos taurus polyomavirus 1
YP 009175001 Desmodus rotundus polyomavirus 1
YP 009174996 Pteronotus parnellii polyomavirus 1
YP 007346978 Pteronotus polyomavirus
YP 007346973 Miniopterus africanus polyomavirus 1
YP 009134726 Sheep polyomavirus 1
YP 009129311 Bat polyomavirus 6c
YP 009129299 Bat polyomavirus 6d
YP 002261488 Myotis polyomavirus VM-2008
YP 009111357 Mastomys natalensis polyomavirus 1
YP 009315927 Polyomavirus sp.
NP 041234 Mus musculus polyomavirus 2
YP 007195276 Cebus albifrons polyomavirus 1
YP 001531348 Squirrel monkey polyomavirus
YP 009351895 Rhinolophus hildebrandtii polyomavirus 1
YP 009129281 Bat polyomavirus 6a
YP 009351901 Rousettus aegyptiacus polyomavirus 1
YP 009129315 Bat polyomavirus 6b
YP 008603285 African elephant polyomavirus 1
Tasmanian devil-associated polyomavirus 1
YP 006495788 MW polyomavirus
YP 007354884 STL polyomavirus
YP 009325930 Rattus norvegicus polyomavirus 2
YP 001285487 WU Polyomavirus
YP 001111258 Human polyomavirus 3
YP 009174987 Common vole polyomavirus
YP 009174981 Bank vole polyomavirus
YP 009352861 Alpaca polyomavirus
YP 009110693 Bovine polyomavirus 3
YP 003848918 Human polyomavirus 6
YP 003848923 Human polyomavirus 7
YP 009116784 Giant guitarfish polyomavirus 1
YP 009345067 Pomona leaf-nosed bat associated polyomavirus
YP 009272696 Sparus aurata polyomavirus 1
YP 009134737 Trematomus polyomavirus 1
YP 009110676 Black sea bass polyomavirus 1
YP 009110674 Black sea bass polyomavirus 1
0.5

Fig. S5

LTAg

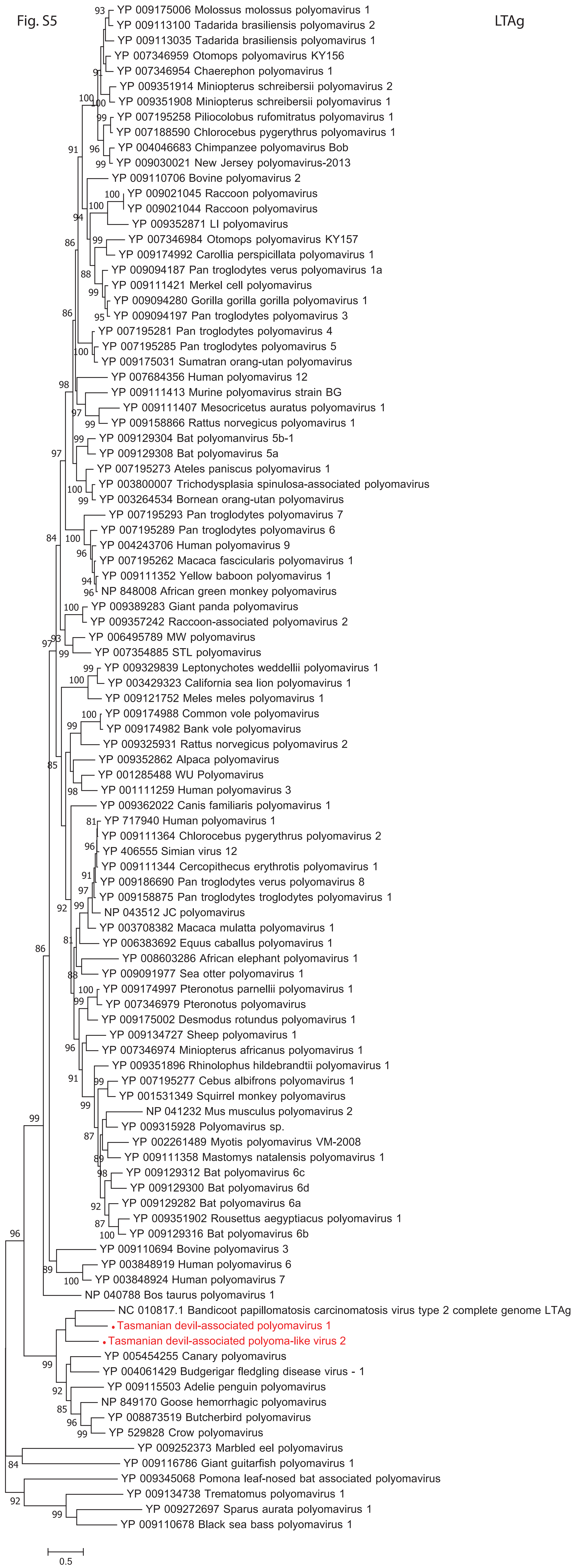
YP 009175006 Molossus molossus polyomavirus 1
YP 009113100 Tadarida brasiliensis polyomavirus 2
YP 009113035 Tadarida brasiliensis polyomavirus 1
YP 007346959 Otomops polyomavirus KY156
YP 007346954 Chaerephon polyomavirus 1
YP 009351914 Miniopterus schreibersii polyomavirus 2
YP 009351908 Miniopterus schreibersii polyomavirus 1
YP 007195258 Piliocolobus rufomitratus polyomavirus 1
YP 007188590 Chlorocebus pygerythrus polyomavirus 1
YP 004046683 Chimpanzee polyomavirus Bob
YP 009030021 New Jersey polyomavirus-2013
YP 009110706 Bovine polyomavirus 2
YP 009021045 Raccoon polyomavirus
YP 009021044 Raccoon polyomavirus
YP 009352871 LI polyomavirus
YP 007346984 Otomops polyomavirus KY157
YP 009174992 Carollia perspicillata polyomavirus 1
YP 009094187 Pan troglodytes verus polyomavirus 1a
YP 009111421 Merkel cell polyomavirus
YP 009094280 Gorilla gorilla gorilla polyomavirus 1
YP 009094197 Pan troglodytes polyomavirus 3
YP 007195281 Pan troglodytes polyomavirus 4
YP 007195285 Pan troglodytes polyomavirus 5
YP 009175031 Sumatran orang-utan polyomavirus
YP 007684356 Human polyomavirus 12
YP 009111413 Murine polyomavirus strain BG
YP 009111407 Mesocricetus auratus polyomavirus 1
YP 009158866 Rattus norvegicus polyomavirus 1
YP 009129304 Bat polyomanvirus 5b-1
YP 009129308 Bat polyomavirus 5a
YP 007195273 Ateles paniscus polyomavirus 1
YP 003800007 Trichodysplasia spinulosa-associated polyomavirus
YP 003264534 Bornean orang-utan polyomavirus
YP 007195293 Pan troglodytes polyomavirus 7
YP 007195289 Pan troglodytes polyomavirus 6
YP 004243706 Human polyomavirus 9
YP 007195262 Macaca fascicularis polyomavirus 1
YP 009111352 Yellow baboon polyomavirus 1
NP 848008 African green monkey polyomavirus
YP 009389283 Giant panda polyomavirus
YP 009357242 Raccoon-associated polyomavirus 2
YP 006495789 MW polyomavirus
YP 007354885 STL polyomavirus
YP 009329839 Leptonychotes weddellii polyomavirus 1
YP 003429323 California sea lion polyomavirus 1
YP 009121752 Meles meles polyomavirus 1
YP 009174988 Common vole polyomavirus
YP 009174982 Bank vole polyomavirus
YP 009325931 Rattus norvegicus polyomavirus 2
YP 009352862 Alpaca polyomavirus
YP 001285488 WU Polyomavirus
YP 001111259 Human polyomavirus 3
YP 009362022 Canis familiaris polyomavirus 1
YP 717940 Human polyomavirus 1
YP 009111364 Chlorocebus pygerythrus polyomavirus 2
YP 406555 Simian virus 12
YP 009111344 Cercopithecus erythrotis polyomavirus 1
YP 009186690 Pan troglodytes verus polyomavirus 8
YP 009158875 Pan troglodytes troglodytes polyomavirus 1
NP 043512 JC polyomavirus
YP 003708382 Macaca mulatta polyomavirus 1
YP 006383692 Equus caballus polyomavirus 1
YP 008603286 African elephant polyomavirus 1
YP 009091977 Sea otter polyomavirus 1
YP 009174997 Pteronotus parnellii polyomavirus 1
YP 007346979 Pteronotus polyomavirus
YP 009175002 Desmodus rotundus polyomavirus 1
YP 009134727 Sheep polyomavirus 1
YP 007346974 Miniopterus africanus polyomavirus 1
YP 009351896 Rhinolophus hildebrandtii polyomavirus 1
YP 007195277 Cebus albifrons polyomavirus 1
YP 001531349 Squirrel monkey polyomavirus
NP 041232 Mus musculus polyomavirus 2
YP 009315928 Polyomavirus sp.
YP 002261489 Myotis polyomavirus VM-2008
YP 009111358 Mastomys natalensis polyomavirus 1
YP 009129312 Bat polyomavirus 6c
YP 009129300 Bat polyomavirus 6d
YP 009129282 Bat polyomavirus 6a
YP 009351902 Rousettus aegyptiacus polyomavirus 1
YP 009129316 Bat polyomavirus 6b
YP 009110694 Bovine polyomavirus 3
YP 003848919 Human polyomavirus 6
YP 003848924 Human polyomavirus 7
NP 040788 Bos taurus polyomavirus 1
NC 010817.1 Bandicoot papillomatosis carcinomatosis virus type 2 complete genome LTAg
Tasmanian devil-associated polyomavirus 1
Tasmanian devil-associated polyoma-like virus 2
YP 005454255 Canary polyomavirus
YP 004061429 Budgerigar fledgling disease virus - 1
YP 009115503 Adelie penguin polyomavirus
NP 849170 Goose hemorrhagic polyomavirus
YP 008873519 Butcherbird polyomavirus
YP 529828 Crow polyomavirus
YP 009252373 Marbled eel polyomavirus
YP 009116786 Giant guitarfish polyomavirus 1
YP 009345068 Pomona leaf-nosed bat associated polyomavirus
YP 009134738 Trematomus polyomavirus 1
YP 009272697 Sparus aurata polyomavirus 1
YP 009110678 Black sea bass polyomavirus 1
0.5
